# Neonatal treatment of CINCA syndrome

**DOI:** 10.1186/1546-0096-12-52

**Published:** 2014-12-15

**Authors:** Yan Paccaud, Gerald Berthet, Annette Von Scheven-Gête, Bernard Vaudaux, Yvan Mivelaz, Michael Hofer, Matthias Roth-Kleiner

**Affiliations:** Clinic of Neonatology, Department of Pediatrics, University Hospital and University of Lausanne, Lausanne, Switzerland; Clinic of Pediatrics, Cantonal Hospital of Aarau, Aarau, Switzerland; Unit of Pediatric Rheumatology of Western Switzerland, Departments of Pediatrics, University Hospital and University of Lausanne, and University Hospital of Geneva, Geneva, Switzerland; Pediatric Infectiology Unit, Department of Pediatrics, University Hospital and University of Lausanne, Lausanne, Switzerland; Pediatric Cardiology Unit, Department of Pediatrics, University Hospital and University of Lausanne, Lausanne, Switzerland; Clinic of Neonatology, Centre Hospitalier Universitaire Vaudois, CHUV, Av Pierre Decker 2, CH-1011 Lausanne, Switzerland

**Keywords:** CINCA, NOMID, Neonatal treatment, Anakinra, IL-1 receptor, IL-1β

## Abstract

**Abstract:**

Chronic Infantile Neurological Cutaneous Articular (CINCA) syndrome, also called Neonatal Onset Multisystem Inflammatory Disease (NOMID) is a chronic disease with early onset affecting mainly the central nervous system, bones and joints and may lead to permanent damage. We report two preterm infants with severe CINCA syndrome treated by anti-interleukin-1 in the neonatal period, although, so far, no experience with this treatment in infants younger than three months of age has been reported. A review of the literature was performed with focus on treatment and neonatal features of CINCA syndrome.

**Case report:**

Two cases suspected to have CINCA syndrome were put on treatment with anakinra in the early neonatal period due to severe clinical presentation. We observed a rapid and persistent decline of clinical signs and systemic inflammation and good drug tolerance. Diagnosis was confirmed in both cases by mutations in the *NLRP3/CIAS1*-gene coding for cryopyrin. As particular neonatal clinical signs polyhydramnios and endocardial overgrowth are to be mentioned.

**Conclusion:**

We strongly suggest that specific treatment targeting interleukin-1 activity should be started early. Being well tolerated, it can be introduced already in neonates presenting clinical signs of severe CINCA syndrome in order to rapidly control inflammation and to prevent life-long disability.

## Background

Chronic Infantile Neurological Cutaneous Articular syndrome (CINCA) (OMIM 607’115), also called Neonatal Onset Multisystem Inflammatory Disease (NOMID), is the most severe form of the Cryopyrin-Associated Periodic Syndromes (CAPS), a continuum of diseases with mutations of the *CIAS1*-gene also called *NLRP3,* coding for cryopyrin. It is clinically characterized by the triad of key symptoms (rash, joint involvement and neurological manifestations) in varying severity due to excessive production of IL-1β [1, 2].

Although the first manifestations of CINCA syndrome may be present at birth, neonatal diagnosis is rare and no experience with specific treatment targeting interleukin-1 activity before three months of age is described in the literature [3–9].

Here we present two preterm cases of severe CINCA syndrome with early diagnosis and neonatal start of treatment with anakinra, in order to avoid long-term sequelae. We also present some additional potential congenital manifestations of the disease.

## Case presentation

### Case report 1

**Figure 1:**
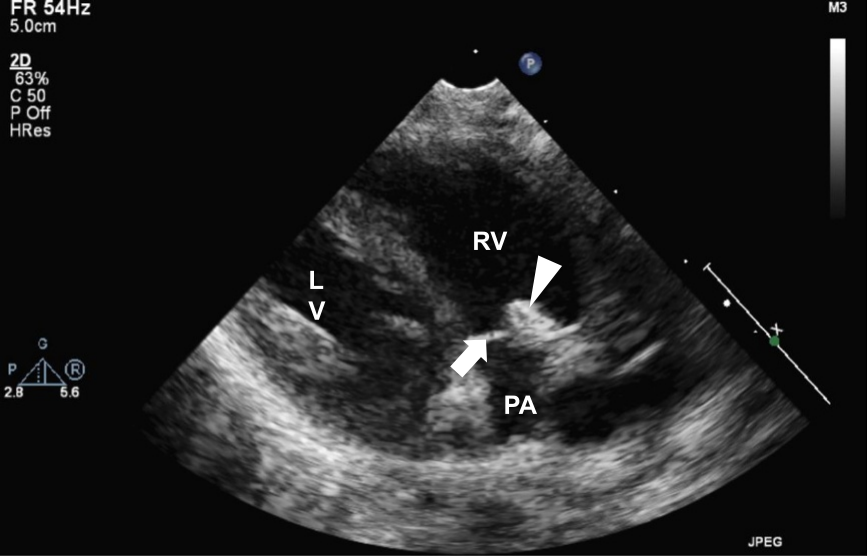
**Cardiac vegetation in neonatal CINCA syndrome.** *Echocardiographic long axis parasternal view of the right ventricular outflow tract*. The pulmonary valve is closed and a thin posterior leaflet is clearly visible (white arrow). The anterior leaflet is thicker and a hyperechogenic lesion (white arrow head) is attached to its ventricular side. (LV: left ventricle; PA: pulmonary artery trunk; RV: right ventricle).

This preterm boy was born at 33 6/7 weeks of gestation after a pregnancy complicated by severe polyhydramnios appearing at 30 weeks of gestation and needing several therapeutic amniocenteses. Extensive search for the aetiology of polyhydramnios was without result. During an amniotic puncture, the patient manifested severe bradycardia, leading to emergency caesarean section. The patient was apnoeic, pale and showed significant hepato-splenomegaly. Resuscitation procedures included positive pressure ventilation, crystalloid and blood administration. The placenta was of augmented weight showing histological signs of umbilical cord infection, but no placental abruption was apparent. Initial laboratory results showed hemoglobin of 71 g/L, leucocytes 52.2×10^9^/L, platelet count 22×10^9^/L, C reactive protein (CRP) 87 mg/L, aspartate aminotransferase 639 U/L, alanine aminotransferase (ALT) 86 U/L, total bilirubin 85 (direct 69) μmol/L. Cerebrospinal fluid (CSF) analysis showed 27×10^6^/L cells (62% neutrophils) and protein 1.19 g/L. Amoxicilline and gentamicine were initiated. A few hours after birth, the patient developed a fluctuating urticarial rash and was febrile during the first few days. Echocardiography showed a lesion similar to infectious vegetation at the pulmonary valve (Figure 1). An extended microbiological evaluation was undertaken: PCR was negative in blood for Parvovirus B19, EBV, in urine for CMV, and in CSF for CMV, Parvovirus and toxoplasmosis. Hepatitis B serology was negative. Serial blood cultures were sterile.

Cerebral MRI was normal except for enlarged pericerebral spaces. Auditory evoked potentials (AEPs) showed unilateral neurosensory deafness (left: 80 dB, right: 40 dB). Ophthalmologic evaluation was normal. Cholestasis which we attributed to parenteral nutrition and the inflammatory process worsened reaching a maximum total bilirubin of 174 μmol/L (direct 147 μmol/L), and ALT 467 U/L. On day 10, the patient developed arthritis of small finger joints, wrists and knees. Antinuclear antibodies and rheumatoid factor were normal; C3 complement factor and serum amyloid A (41.8 mg/L) were elevated. CINCA syndrome was diagnosed based on the symptoms and the absence of infection. After discussion with the parents and their consent, treatment with anakinra (2 mg/kg/day, sc) was initiated at day 17. Already after three injections the rash disappeared, CRP reached normal values, and the arthritis improved. However, as the existing literature about anakinra had mentioned that higher doses were needed in young infants (>3 months) with up to 10 mg/kg/d, we increased the dose stepwise up to 7 mg/kg/d in order to get the patient free of any sign of arthritis [5]. Thrombocytopenia resolved but anaemia persisted for 8 weeks. The endocardial lesion of the pulmonary valve was still present at 8 weeks, however, disappeared without specific treatment by 6 months. Cholestasis slowly resolved in parallel to the decreasing inflammatory reaction and due to treatment with ursodeoxycholic acid and semi-elementary diet. After three weeks of treatment with anakinra, an acute urinary tract infection (UTI) due to Escherichia coli was treated by antibiotics. Anakinra dose was transitorily decreased, whereas today rather an increase of the dose is suggested [4]. After 6 months of treatment, anakinra was reduced to 3 mg/kg/day and the disease remained inactive. At 12 months, neurological evaluation was normal and AEPs showed a moderate neurosensory deafness on both sides (left: 65 dB; right: 50 dB). Ophthalmologic evaluation remained normal at 1 and 2 months. Genetic testing exhibited a heterozygote mutation E567K (p.GLU567Lys) of the *NLRP3* gene [10].

### Case report 2

**Figure 2:**
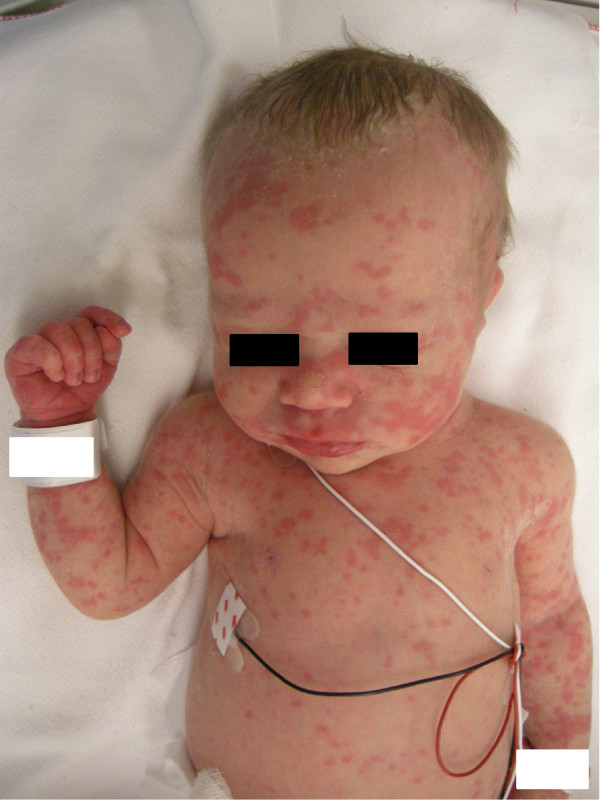
**Cutaneous rash in neonatal CINCA syndrome.**

**Figure 3:**
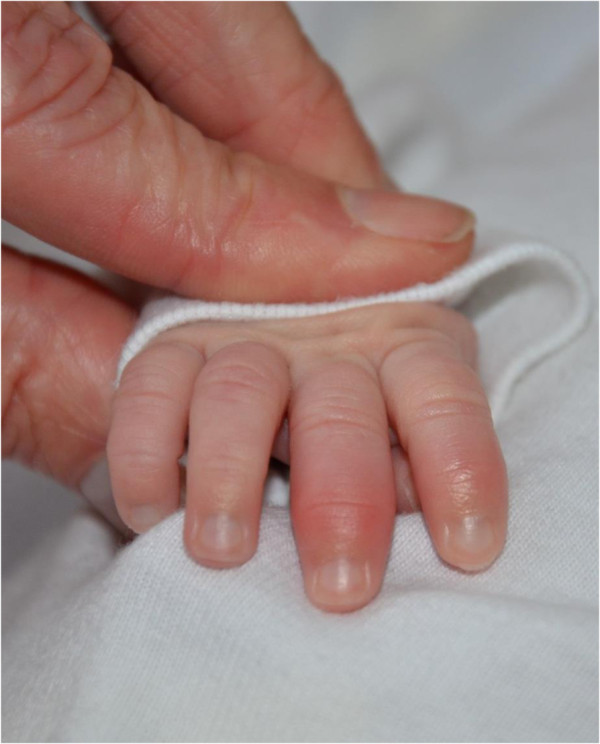
**Arthritis in neonatal CINCA syndrome.**

Born vaginally at 34 4/7 weeks of gestation (birth weight 2650 g), this girl was transferred after normal adaptation to the tertiary centre because of a cutaneous rash starting 7 hours after birth for suspicion of sepsis. She was afebrile, stable cardio-respiratory-wise with a normal neurological status. Urticarial rash was covering her whole body (Figure 2). Blood workup showed normal formula and blood gas values, but increased inflammation parameters (CRP 101 mg/L, Interleukin-6 528 ng/L). CRP raised (137 mg/L) despite initiation of amoxicillin, gentamicine and fluconazole intravenously. Because of sterile blood cultures and absence of clinical signs of infection, the antibiotics were stopped after 4 days. CSF showed 36×10^6^/L leucocytes and cultures were sterile. Cerebral and abdominal ultrasonographies were normal. On day six she developed arthritis in small joints of hands (Figure 3) and feet. Arthritis, together with the rash and the persisting elevated inflammation markers led to the diagnosis of CINCA syndrome. The patient was discharged home with decreasing urticarial rash but persistent arthritis. On day 32, the girl developed fever (38.2°C), with CRP rising to 222 mg/L. This severe evolution prompted parents to accept treatment with anakinra at 39 weeks postmenstrual age. The goals of this treatment were to get the patient free of symptoms and to suppress the inflammatory reaction and reaching normal CRP values. To achieve these goals the dose had to be increased temporarily to 20 mg/kg/d which was well tolerated. At 2 months, therapy was switched to canakinumab, 8 mg/kg sc, every 5–6 weeks without symptoms or increase of inflammatory parameters [11]. At 20 months, neurodevelopment, brain MRI, AEPs and ophthalmologic examination were normal. In the *NLRP3*-gene a heterozygous mutation for F566L (p.Phe566Leu) was identified [10].

## Discussion

CINCA syndrome is a chronic disease with onset in the neonatal period. A severe course affecting mainly the central nervous system, bones and joints, may lead to permanent damage in these organs. Since the early 2000s, a specific treatment, able to control systemic inflammation is available [4]. Therefore, early diagnosis is warranted to start treatment before the occurrence of sequelae induced by the inflammatory process.

Both patients were diagnosed in the neonatal period based on the classical clinical features, and after exclusion of a neonatal infection. Often associated to CINCA syndrome are prematurity, intrauterine growth retardation and oligohydramnios. In addition to the typical symptoms, case 1 presented with severe anaemia and thrombocytopenia, cholestasis, endocardial lesion and polyhydramnios. Anaemia and associated hepato-splenomegaly are well described in CINCA patients [12]. Thrombocytopenia, so far not associated, might be related to the severe anaemia with increased erythropoiesis due to diversion of hematopoietic stem cells away from platelet formation [12, 13]. Macrophage activation syndrome was excluded by normal levels of ferritin and triglycerides. Whereas in recent literature oligohydramnios in CINCA patients has been linked to placenta insufficiency, polyhydramnios was suggested to be a sign of severity of prenatal involvement [14]. The cardiac lesion in the absence of infection is a unique finding and might represent a pathological overgrowth of cells due to the deregulation of the inflammasome, similar to the fibrous dysplasia described in knees of NOMID patients [15]. To exclude a process corresponding to Libman-Sacks endocarditis described in patients with systemic lupus, we searched for antinuclear antibodies which were negative [16].

CAPS are associated with gain-of-function mutations of the *NLRP3/CIAS1*-gene, coding for cryopyrin [1, 17]. Until now, there are more than 170 mutations described [18]. The two mutations found in the here presented patients have been found in other patients: the E567K mutation in a 26 year old woman with Muckle Wells syndrome, another form of CAPS, and the F556L mutation in a 5 year old boy with CINCA syndrome leading to motor developmental delay, mild conductive hearing loss and focal cortical dysplasia with epileptic seizures [19–21]. Due to a wide range of phenotypic heterogeneity, phenotype and mutations do not match well [7, 10, 22]. Moreover, only in half of typical CINCA patients a mutation in the *NLRP3* gene could be found by conventional sequencing. Further subcloning and sequencing of *NLRP3* may detect a somatic *NLRP3* mosaicism in primary genetic-negative CINCA patients [10]. Therefore, the clinician should rely on the clinical features to assess the diagnosis, in particular in young infants.

Due to the central role of excessive IL-1β activity, the contemporary treatment consists in competitively inhibiting IL-1 receptor (anakinra), in diminishing IL-1 activity by IL-1 binding protein (rilonacept) or human anti-IL-1β monoclonal antibody (canakinumab) [1]. Treatment with anakinra was shown to be safe but has not yet been used at neonatal age [3–5, 23]. Anakinra has recently been approved for use in children aged ≥8 months. Because of its short half-life of a few hours, anakinra is the treatment of choice in patients in which the drug tolerance has not been clearly established, e.g. newborn infants and paediatric patients. In regards to the potentially severe sequelae of the disease, the safety of the anti-IL-1 treatment, and the limitations of the genetic results due to phenotypic heterogeneity and frequent mosaicism, genetic testing is not required for starting to use anakinra [4, 5, 7]. According to the literature, patients diagnosed in younger age needed higher doses of anakinra and required more frequent treatment adjustments than adults [4, 5]. Except for an UTI in Case 1, neither patient showed any of the known side effects or complications of the treatment, like injection site reaction, infections particularly by encapsulated-germs, influenza-like symptoms, nausea, diarrhoea, or neutropenia.

The long-term outcome of patients with CINCA syndrome depends on the severity and duration of the inflammatory process. Hearing loss reversibility seems to be correlated with early treatment [1, 2, 4]. It is suggested that complications may be prevented if adequate and efficient treatment is started before the appearance of irreversible lesions [23].

## Conclusion

The clinical triad of cutaneous rash, arthritis and meningitis-like symptoms, associated with severe inflammation without infection should point, already in newborn infants, towards the diagnosis of CINCA syndrome. These two cases suggest that, in severe situations, treatment can be initiated safely in neonates in order to prevent irreversible organ damage and life-long disability. Results of genetic testing are not required before initiation of treatment, as in many cases conventional sequencing for *NLRP3* mutations is negative.

## Consent

Written informed consent was obtained from the patients’ parents for publication of these cases and any accompanying images. A copy of the written consent is available for review by the Editor-in-Chief of this journal.

## Authors’ information

Yan Paccaud and Gerald Berthet equal contribution as first authors.

Matthias Roth-Kleiner and Michael Hofer equal contribution as last authors.

## Abbreviations

CINCA: Chronic Infantile Neurological Cutaneous Articular
NOMID: Neonatal Onset Multisystem Inflammatory Disease
CAPS: Cryopyrin-Associated Periodic Syndromes
CRP: C reactive protein
ALT: Alanine aminotransferase
CSF: Cerebrospinal fluid
PCR: Polymerase chain reaction
EBV: Epstein Barr virus
CMV: Cytomegalo virus
AEP: Auditory evoked potentials
UTI: Urinary tract infection.
